# TRPM4 non-selective cation channel variants in long QT syndrome

**DOI:** 10.1186/s12881-017-0397-4

**Published:** 2017-03-18

**Authors:** Thomas Hof, Hui Liu, Laurent Sallé, Jean-Jacques Schott, Corinne Ducreux, Gilles Millat, Philippe Chevalier, Vincent Probst, Romain Guinamard, Patrice Bouvagnet

**Affiliations:** 1 Normandie University, UNICAEN, EA 4650, Groupe Signalisation, Electrophysiologie et Imagerie des Lésions d’Ischémie-Reperfusion Myocardique, F-14032 Caen, France; 2 Laboratoire Cardiogénétique, Institut de Biologie et Chimie des Protéines, INSERM UMR 5305, Université Lyon 1, Lyon, France; 3 Laboratoire Cardiogénétique, Centre de Biologie et Pathologie Est, Hospices Civils de Lyon, Bron, France; 4 Institut du thorax, INSERM UMR 1087, CNRS UMR 6291, Nantes, France; 5 Service de Cardiologie Pédiatrique, Hôpital Louis Pradel, Bron, France; 6 Laboratoire Cardiogénétique Moléculaire, Centre de Biologie et Pathologie Est, Hospices Civils de Lyon, Bron, France; 7 Service de Cardiologie, Hôpital Louis Pradel, Bron, France; 8 Institut du thorax, Service de Cardiologie, CHU Nantes, Nantes, France; 9 Present Address: Department of Anatomy, Hainan Medical College, Haikou, 571101 Hainan China; 10 Laboratoire Cardiogénétique, Groupe Hospitalier Est, 59 boulevard Pinel, CBPE, 69677 Bron, France

**Keywords:** Long QT syndrome, TRPM4, Electrophysiology, Outward rectifying current, Arrhythmia, Gene mutation

## Abstract

**Background:**

Long QT syndrome (LQTS) is an inherited arrhythmic disorder characterized by prolongation of the QT interval, a risk of syncope, and sudden death. There are already a number of causal genes in LQTS, but not all LQTS patients have an identified mutation, which suggests LQTS unknown genes.

**Methods:**

A cohort of 178 LQTS patients, with no mutations in the 3 major LQTS genes (*KCNQ1*, *KCNH2*, and *SCN5A*), was screened for mutations in the transient potential melastatin 4 gene (*TRPM4*).

**Results:**

Four *TRPM4* variants (2.2% of the cohort) were found to change highly conserved amino-acids and were either very rare or absent from control populations. Therefore, these four *TRPM4* variants were predicted to be disease causing. Furthermore, no mutations were found in the DNA of these TRPM4 variant carriers in any of the 13 major long QT syndrome genes. Two of these variants were further studied by electrophysiology (p.Val441Met and p.Arg499Pro). Both variants showed a classical TRPM4 outward rectifying current, but the current was reduced by 61 and 90% respectively, compared to wild type TRPM4 current.

**Conclusions:**

This study supports the view that *TRPM*4 could account for a small percentage of LQTS patients. *TRPM4* contribution to the QT interval might be multifactorial by modulating whole cell current but also, as shown in Trpm4^−/−^ mice, by modulating cardiomyocyte proliferation. *TRPM4* enlarges the subgroup of LQT genes (*KCNJ2* in Andersen syndrome and *CACNA1C* in Timothy syndrome) known to increase the QT interval through a more complex pleiotropic effect than merely action potential alteration.

## Background

Congenital long QT syndrome (LQTS) is characterized by a prolonged QT interval on the electrocardiogram (ECG), and can lead to cardiac events such as syncope or sudden cardiac death due to torsade de pointes or ventricular fibrillation. Prevalence in the population is about 1 over 2000 to 3000 [1, 2].

Similarly to other cardiomyopathies, a number of genes were described to be responsible for LQTS. To date, 15 genes have already been identified with more than 700 mutations in LQTS patients [3, 4]. Six of these genes encode for pore-forming ion channel subunits, while others encode for regulatory subunits or proteins. Among ion channel genes, mutations in *KCNQ1* and *KCNH2*, which encode voltage-gated K^+^ channels involved in cardiac action potential (AP) repolarization, are responsible for about 70% of LQTS [3]. Mutations of the voltage-gated Na^+^ channel encoded by the *SCN5A* gene are responsible for 1.7 to 8% of LQTS while mutations in other genes are rare (below 1% of LQTS). Altogether, the quest to find mutations remains elusive in about 25% of LQTS patients [5], which leaves place for the discovery of additional causal genes for this syndrome.

At the mechanistic level, LQT is mainly explained by a delayed ventricular repolarization due to a prolongation of AP in ventricular cardiomyocytes. It explains why loss of function mutations in voltage-gated K^+^ channel encoding genes, such as *KCNQ1* and *KCNH2*, which participate in cardiomyocyte repolarization (AP phases 2–3), result in LQTS. Similarly, any ion channel known to participate in the shape of AP may be involved in LQTS. Several clues pointed to the transient potential melastatin 4 gene (*TRPM4*) as a pertinent contender for LQTS: 1- *TRPM4* encodes a non-selective cation channel and the mRNA is present in the heart [6]. 2- TRPM4 currents were recorded in cardiomyocytes of several mammals including humans [7]. 3- *Trpm4* ^*−/−*^ mice have an increased QT interval [8]. 4- *TRPM4* mutations were reported in patients with other cardiac electrical disturbances such as, cardiac conduction block [9, 10] and Brugada syndrome [11]. The involvement of *TRPM4* in several pathologies is reminiscent to the voltage-gated Na^+^ channel encoded by *SCN5A*, which was associated with LQTS, Brugada syndrome, and conduction blocks [12].

According to this, we hypothesized that *TRPM4* variants might be linked to LQTS. To address this hypothesis, we screened a cohort of 178 patients with LQTS and had no identified mutations in the major LQTS genes (*KCNQ1*, *KCNH2* and *SCN5A*). *TRPM4* variants were found in 4 patients (2.2% of the cohort). The electrophysiological properties of 2 of these mutants were further studied. Both mutations resulted in a decrease of function of the TRPM4 channel.

## Methods

### Study population

In this study, 178 patients from the University Hospitals of Nantes and Lyon were tested. This study was approved by the local ethics committees (*comité de protection des personnes Ouest IV* and *Sud-Est II*) and is in accordance with the last version of the Declaration of Helsinki [13]. In all probands, the clinical diagnosis of LQTS was made using the Schwartz criteria [14, 15]. The QT interval was corrected by the formula of Bazett (QTc). Each patient underwent full medical examination to rule out syndromic forms of LQT. Blood samples were collected after written informed consent. All study participants provided written informed consent to participate to this study. All these probands were negative after mutation screening by dHPLC or direct sequencing in the *KCNQ1*, *KCNH2*, and *SCN5A* genes. The LQTS cohort of the University Hospital of Nantes (90 patients) had also been tested negative for copy number variations with a Multiple Ligation-dependent Probe Amplification test of MRC Holland (SALSA P114 MLPA, MRC-Holland, Amsterdam, the Netherlands). This test contains 20 probes interrogating the *KCNQ1* gene, 9 for the *KCNH2* gene, and 3 for the *SCN5A* gene [15]. The LQTS cohort of the University Hospital of Lyon (88 patients) was also negative for mutation in the *KCNE1* and *KCNE2* genes.

### Genetic analysis

DNA was extracted from blood samples according to standard protocols. DNA concentration and quality were assessed using NanoDrop (Thermo Fisher Scientific, Villebon sur Yvette, France) and Qubit (Life technologies, Villebon sur Yvette, France) fluorometers. A260/A280 ratios of 1.8 to 2.0 and A260/A230 ratios >1.5 were accepted. Mutation screening of *TRPM4* (ENST00000252826, NM_017636.3, human genome version GRCh37/hg19) was carried out by High Resolution Melting (HRM) analysis (Rotor-Gene Q, Qiagen, Courtaboeuf, France), followed by bi-directional sequencing of abnormal profiles or by direct sequencing. The primers were already published [10]. Variants were confirmed on a second sample and PCR product. For miniexome sequencing, enrichment of DNA samples was performed with the Nextera Rapid Capture Exome v1.0 kit (Illumina, San Diego, CA, USA). Sequencing was carried out on a NextSeq500 instrument (Illumina, San Diego, CA, USA) with a high-output flow cell. TruSight One targets 4813 clinically relevant genes and covers 12 Mb of genomic content. In particular, the following long QT genes are included: *ANK2*, *CACNA1C*, *CALM1*, *CAV3*, *KCNE1*, *KCNE2*, *KCNJ2*, *KCNJ5*, *KCNQ1*, *SCN4B*, *SCN5A* and *SNTA1*. A minimal coverage of 10x was obtained on 98.2% of target sequences. Bam files were loaded on Alamut Visual 2.7.1 (Interactive Biosoftware, Rouen, France) and the 13 long QT genes were sequentially visualized along with the sequence reads of each patient. A threshold of 30% was selected to highlight sequence variants. The coverage of all coding exons of LQ long genes was at least 10x.

### Preparation of TRPM4 mutants

The complete human wild-type *TRPM4* cDNA was cloned in a pcDNA4/TO vector (Invitrogen, Cergy Pontoise, France) [10]. Mutants were obtained by in vitro mutagenesis using QuickChange II site-directed mutagenesis kit (Agilent Technologies, Massy, France). Mutant cDNA clones were then systematically resequenced before using them in further experiments.

### Stable TRPM4 mutant expression

PcDNA4/TO plasmid containing the 2 selected *TRPM4* mutants, were used to transfect T-REx™ 293 cell lines with Lipofectamine 2000 (Invitrogen, Cergy Pontoise, France) according to manufacturer specifications. The T-REx™ 293 cell line stably expresses the tetracycline repressor protein, enabling the silencing of the gene of interest unless tetracycline is added to the culture medium. T-REx™ 293 was obtained by stably transforming HEK 293 cells with a plasmid containing the Tet repressor cDNA under the control of the human CMV promoter. Several stable clones (3–4) of each *TRPM4* mutants were obtained according to Invitrogen protocol, by selecting with blasticidin (Tet repressor) and zeocin (TRPM4). These stable clones were used for the electrophysiological study.

### Electrophysiological recordings

Patch-clamp experiments on T-REx™ 293 transfected cells were performed at room temperature. An Axopatch 200B (Axon Instruments, Sunnyvale, CA, USA) amplifier was used and controlled by a Pentium PC connected by a Digidata 1322A A/D converter (Axon Instruments). It was also used for data acquisition and analysis using Pclamp software (Axon Instruments). Signals were filtered at 1 kHz using an 8-pole Bessel low pass filter before digitization at 10 kHz and storage. Patch pipettes resistance was typically 2 MΩ when filled with intracellular solution (below).

TRPM4 currents were recorded in the whole-cell configuration using a ramp protocol. The holding potential was −60 mV. The 400 ms increasing ramp from −100 to +100 mV ends with a 20 ms step at +100 mV to unmask a typical TRPM4 current [16]. The measured current was then reported to cell size estimated by capacitance measurement. A new ramp was performed every 5 s. TRPM4 current develops with time after the membrane break to stabilize within 10–20 min as previously shown [16]. Biophysical properties were estimated after current stabilization. Pipette solutions contained (in mM) 156 CsCl, 1 MgCl2 and 10 HEPES (pH adjusted to 7.2 with CsOH and [Ca^2+^] 10^−4^ M). Bath and perfused solutions contained (in mM) 156 NaCl, 5 CaCl2, 10 glucose and 10 HEPES (pH adjusted to 7.4 with NaOH).

### Data analysis

Mutant electrophysiological parameters were compared to wild type using an unpaired Student *t* test, with a probability value below 0.05 considered as significant. A Fisher exact test was performed to compare the prevalence of *TRPM4* variants in the ethnically matched control population, and in the LQTS cohort both of European origin.

## Results

### Study subjects and *TRPM4* screening

The screening of *TRPM4* in the cohort of 178 Long QT cases with no mutations in the 3 major LQT genes (*KCNH2*, *KCNQ1*, and *SCN5A*) evidenced 4 variants of interest (Table 1): p.V441M, p.R499W, p.R499P, and p.G844D. The putative pathogenicity of these variants was estimated on their prevalence in ethnically matched control populations, the interspecies conservation of reference amino acid, the physico-chemical gap between referenced and new residues, and prediction software (snp&go [17], PhD_SNP [18], SNAP [19] and PredictSNP1.0 [20]). The data is summarized in Table 1. All variants were new [9–11, 21] except p.G844D, which was already found in cases of conduction blocks [10, 21] and of Brugada syndrome [11]. All variants were heterozygous. To exclude that any of these 4 Patients had a variant in an already known long QT genes, the sequencing of a panel of 4813 clinically relevant genes was obtained from each patient. A careful reading with Alamut Visual 2.7.1 of sequence reads of each *TRPM4* variant carriers did not detect any suspicious variant in coding exons of any of the 13 long QT genes (*ANK2*, *CACNA1C*, *CALM1*, *CAV3*, *KCNE1*, *KCNE2*, *KCNH2*, *KCNJ2*, *KCNJ5*, *KCNQ1*, *SCN4B*, *SCN5A* and *SNTA1*) tested with TruSight One gene panel. Interestingly, this gene panel includes the *TRPM4* gene. It was thus possible to confirm the reported *TRPM4* variant of each of the 4 Patients.

**Table 1:** Variant characteristics

Patient ID	cDNA	Exon	Protein	Local controls	EVS Eur-Am	ExAC Eur-Am	Combined controls	Fisher exact test	Interspecies conservation	Grantham score	snp&go	PhD-SNP	SNAP Prediction	SNAP Expected Accuracy	Predict SNP1.0
1	c.1321 G > A	11	p.V441M	0/551	A = 0/G = 8600	A = 6/G = 66466	6/82637	0.015*	12/12	21	Disease (5)	Disease (7)	Non-neutral	78%	Deleterious (76%)
2	c.1495 C > T	11	p.R499W	1/410	T = 2/C = 8588	T = 12/C = 62148	15/78160	0.036*	6/12	101	Neutral (1)	Disease (3)	Non-neutral	70%	Deleterious (87%)
3	c.1496 G > C	11	p.R499P	0/410	C = 0/G = 8588	C = 0/G = 62148	0/78160	0.002*	6/12	103	Disease (5)	Disease (7)	Non-neutral	63%	Neutral (73%)
4	c.2531 G > A	17	p.G844D	4/1000	A = 13/G = 8359	A = 37/G = 8324	55/18440	0.417	7/9^a^	94	Neutral (7)	Neutral (4)	Non-neutral	78%	Neutral (75%)

Combined controls: total of local controls and all European controls of EVS and ExAC databases

*Abbreviations*: *EVS Eur-Am* Exome Variant Server European American population, *ExAC* Exome Aggregation Consortium

*p < 0.05

^a^not conserved in *Platypus* and *Fugu*

The clinical details of the 4 variant carriers are presented in Table 2. The range of age of discovery was wide from 3 months to 69 years of age. Circumstances of discovery were essentially related to disturbance in consciousness.

**Table 2:** Patients characteristics

Patient ID	Protein	Sex	Age at diagnosis	Circumstances of discovery	Family history	Heart rate	PR interval	Conduction block	QT interval	QTc interval	Other cardiac	LQT score
1	p.V441M	M	64 y	ECG	No	56	178	LAHB	560	539	Notched T waves, Syncope, Exercise dyspnea	4
2	p.R499W	M	3 m	4 Fainting episodes	No	86	120	None	394	471	-	3.5
3	p.R499P	F	69 y	Fainting	No	68	140	None	440	469	Ventricular arrhythmia	3.5
4	p.G844D	M	15 y	Resuscitated SCD	Yes	58	134	None	498	490	-	4.5

*Abbreviations*: *ECG* electrocardiogram, *LAHB* Left Anterior HemiBlock, *mo* month, *QTc* corrected QT interval (Bazett formula), *SCD* resuscitated Sudden Cardiac Death, *y* year

**Fig. 1:**
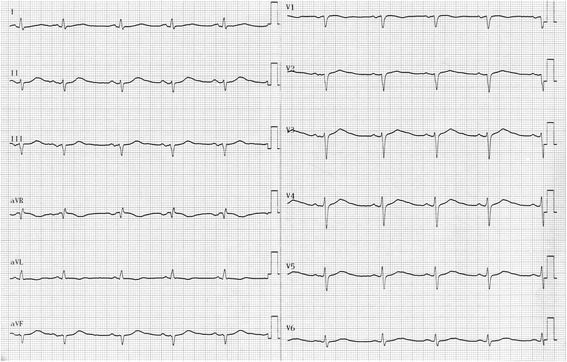
ECG of Patient 1: This patient was 70 years old at the time of ECG. He had a left anterior hemiblock and a QTc interval at 539 ms (QT 560 ms and heart rate 56/min)

*Patient 1* had hypertension, type II diabetes and was over-weighted. He was first seen at the cardiology clinics because he was complaining about a shortness of breath during exercise. He had a normal echocardiography and normal coronarography but a prolonged QTc interval was discovered. He was given 20 mg daily of Netaxolol. Four years later, he was 68 years old. He lost consciousness for several minutes while at rest. His wife noticed that he had neither seizures nor urine loss. He recovered consciousness spontaneously. Upon arrival of the emergency unit, his heart rate was low (30/min) with numerous premature ventricular beats occurring at the down-sloping part of the T wave and a QTc at 500 ms. However, no torsade-de-pointes were recorded during his monitoring. He had hypokaliemia (2.8 mEq/L) although he did not take any diuretic. He received potassium and the dosage of betablocker was lowered. Two years later, he started developing a mild cardiac failure and his ECG showed, in addition to the long QT, a left anterior hemiblock (Fig. 1). His echocardiogram showed a mild concentric hypertrophy (10 mm), a normal ejection fraction and a diastolic dysfunction. We consider a Schwartz score of 4 for this patient (QTc ≥ 480 ms and syncope at rest).

**Fig. 2:**
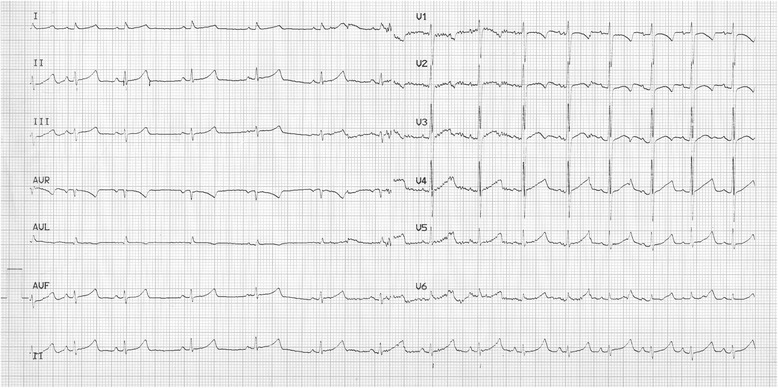
ECG of Patient 2: The boy was 9 years old at the time recording of this ECG. He had a heart rate at 86/min, QT 394 ms and QTc 471 ms as measured automatically

*Patient 2* was a three-year-old baby when he had his first loss of consciousness. As he was sleeping, he had a cry. The parents who rushed to his bed noticed that he was unreactive and atonic for several minutes. Then, he recovered progressively. He was pale with circumoral cyanosis and he vomited. At hospital, he had an echocardiography which disclosed a patent foramen ovale, a prolonged QTc interval. A pH-metry showed no evidence for a gastro-oesophageal reflux. Blood analysis was in normal range. He had an ambulatory ECG which showed no arrhythmia. Subsequently, he had 3 losses of consciousness in the following year with no detection of any other anomalies except mild hypothermia (35.6 °C). Figure 2 presents one representative ECG. We gave a Schwartz score of 3.5 to this patient (QTc between 460 and 479 ms, syncope at rest and a slow heart rate for his age). His asymptomatic older sister and parents had a normal QTc interval.

**Fig. 3:**
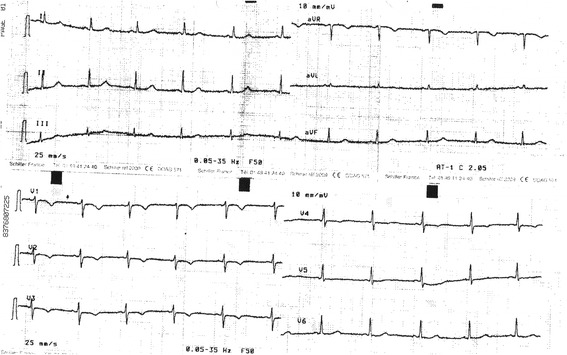
ECG of Patient 3: This 69-year-old female patient had a heart rhythm at 68/min, QT 440 ms and QTc 469 ms

**Fig. 4:**
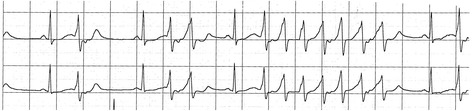
Holter ECG of Patient 3: This recording showed a short bout of ventricular arrhythmia occurring the afternoon of her morning syncope

*Patient 3* is a 69-year-old female who lost consciousness while gardening. She recovered consciousness after an unknown lap of time and felt several episodes of dizziness during the following hours. Her ECG showed a prolonged QT interval (Fig. 3) and Holter recording discovered short stretches of ventricular beats (Fig. 4). Her echocardiogram was normal and her blood tests were normal. She was given a regular regimen of betablocker and did not experience any other episodes of loss of consciousness. In our opinion, her Schwartz score is 3.5 (QTc in the range of 460–479 ms or torsade-like arrhythmia and effort-associated syncope).

**Fig. 5:**
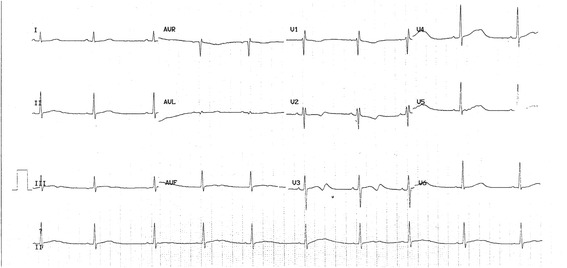
ECG of Patient 4: This boy had a heart rate at 58/min, QT 498 ms and QTc 490 ms

*Patient 4*, a fifteen-year-old boy, had a syncope while he was at rest. The next day at the hospital, his ECG showed a prolonged QTc interval but no arrhythmia (Fig. 5). He had a rSr’ QRS complex in V2 but the QRS duration was normal (90 ms). His echocardiography was normal. His Schwartz score is 4.5 (QTc ≥ 480 ms, syncope and a familial case of unexplained sudden death).

It was not possible to obtain blood samples from relatives of these patients.

### TRPM4 currents in the whole-cell configuration

HEK-293 *TRPM4* transfected cells were searched for ion currents using patch-clamp recordings in the whole-cell configuration. Wild type (WT) *TRPM4* transfected cells exhibited a characteristic outward rectifying current with a density measured at +100 mV of 191 ± 25 pA/pF (*n* = 18), significantly higher than non-transfected HEK-293 cells (3 ± 1 pA/pF; *n* = 11, *p* < 0.001), which is in accordance with previous reports [11, 16].

**Fig. 6:**
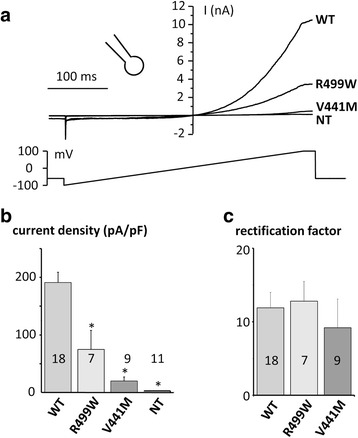
Biophysical properties of currents from HEK-293 cells transfected with WT and TRPM4 variants. **a** Representative current tracings recorded in the whole-cell configuration (ramp protocol under the traces) (**b**) Mean current densities for WT and variants estimated using the maximal current recorded during the ending step of 20 ms at Vm = +100 mV (see **a**). **c** Rectification factor estimated by the current amplitude at +100 mV divided by the current amplitude at −60 mV . *p < 0.05, Error bar: standard error of the mean. Number of experiments is indicated in bars. Abbreviations: NT: Non-Transfected; WT: Wild-type

Both, Val441Met and Arg499Trp, produced an outward rectifying current of 74.6 ± 33 pA/pF (*n* = 7) and 20 ± 7 pA/pF (*n* = 9) respectively, which is significantly higher than the untransfected cells (*p* = 0.014 and *p* = 0.020, respectively) but smaller than WT transfected cells (*p* = 0.016 and *p* < 0.001, respectively) (Fig. 6a and b). To characterize the current rectification, the current at +100 mV was expressed as a ratio to the current at −60 mV (Fig. 6c). No significant variation was induced by mutations when compared to WT, indicating that this parameter was not affected.

## Discussion

Four coding variants, which occurred in less than 0.3% of more than 18,000 control chromosomes (and less than 0.02% of more than 78,000 control chromosomes, if G844D is excepted), were identified in 178 LQT cases with no mutations in the 13 major LQTS genes. Among the genes associated with long QT syndrome, only *AKAP9* and *CALM2* are not included in the clinical exome of Illumina. *AKAP9* was associated with a single case of long QT syndrome [22] and might rather be a QT interval modifier than a long QT causal gene [23]. *CALM2* mutations may result in neonatal bradycardia, cardiac arrest at a young age, a markedly prolonged QTc interval and eventually mild neurodevelopmental delay [24, 25]. Only two patients of this *TRPM4* study were within the age range of *CALM2* mutations (Patients 2 and 4) but their QTc interval was never markedly prolonged as in *CALM2* mutations.

The *TRPM4* variants of this study are all heterozygous missense variants. Interestingly, one patients had in addition to LQTS, a conduction block (patient 1 had a Left Anterior Hemiblock) but he was 70 years old when the heart block appeared. This is consistent with Trpm4^−/−^ mice, which also present multilevel conduction blocks [8] in addition to increased QT interval. The variant of patient 4 (p.G844D) was found in other cases with conduction blocks [10, 21] or Brugada syndrome [11]. Among these latter cases, one also had a prolonged QTc interval of 458 ms (patient 13 of reference 11).

In this study, comparing whole-cell currents recorded from WT and Val441Met and Arg499Trp *TRPM4* transfected HEK293 cells, showed densities significantly lower in both variants. The present study does not discriminate between a decrease in current density due to a modification of biophysical/regulatory properties of the ion channel, or a decreased protein expression as previously reported for other *TRPM4* mutants [11]. However, there is a clear channel loss of function at the level of the membrane.

Until now, TRPM4 impact on cardiac activity was investigated only with regards to its contribution to the action potential (AP). According to its non-selective cation permeability (Na^+^ and K^+^), its opening is suspected to induce an outward repolarizing current in positive voltages but an inward depolarizing current in negative voltages. Since channel activity increases with membrane depolarization, it is suspected to be largely open at the upstroke and during the plateau of the AP. This is potentiated by the fact that TRPM4 is activated by internal Ca^2+^, which increases during the AP. Altogether, one can predict that TRPM4 activation reduces phase 2 duration of AP and prolongs phase 3 counteracting repolarization. It seems that the effect on phase 2 is predominant, at least in mouse atria, since TRPM4 pharmacological inhibition by 9-phenanthrol reduces AP duration [26]. Consistent with this result, Trpm4^−/−^ mice exhibit a shortened AP in atria, but also in ventricle [26, 27]. Surprisingly, ECGs from Trpm4^−/−^ mice showed a prolonged QT interval, despite the reduction of ventricular AP duration [8, 27]. The rationale for this counter-intuitive change might be that *Trpm4* ^*−/−*^ mice also show an increased heart/body weight ratio, which is not due to cardiomyocyte hypertrophy but rather neonatal hyperplasia [8]. QT duration depends on both ventricular AP duration and AP propagation within the ventricle. Thus, hyperplasia could increase the time for the excitation to reach the entire ventricles, and thus prolong the QT interval. Even if AP parameters are different between mice and human, the model of *Trpm4* ^−/−^ mice might be representative of heart disease related to *TRPM4* mutations in human. Indeed, mutations which induce loss of function may impact cardiac development as does *Trpm4* disruption in mice. *TRPM4* is not the first gene encoding a channel whose mutations result in LQT and developmental anomalies as it was already found in Andersen [28] and Timothy [29] syndromes. Unfortunately, contribution of the TRPM4 current to these phases of the AP was not evaluated yet in human. It was also not possible to estimate a putative cardiomyocyte hyperplasia in patients of this study. At least, it can be stated that none of the five TRPM4 variant carriers fulfilled the echographic criteria for hypertrophic cardiomyopathy.

## Conclusion

This study supports the view that *TRPM4* variants could be responsible for about 2% of LQT syndrome cases. The impact of these variants might result not only in electrophysical disturbances, but putatively also in other anomalies including cell proliferation.

## Abbreviations

AP: Action potential
cDNA: copy Deoxyribonucleic acid
CMV: CytoMegalovirus
dHPLC: denaturating High Performance Liquid Chromatography
ECG: Electrocardiogram
Euro-Am EVS: European-American Exome Variant Server
ExAC: Exome Aggregation Consortium
HEK: Human Embryonic Kidney
HRM: High Resolution Melting
LAHB: Left anterior hemiblock
LQTS: Long QT syndrome
MLPA: Multiple Ligation-dependent Probe Amplification
mRNA: messenger Ribonucleic acid
PCR: Polymerase Chain Reaction
QTc: Corrected QT interval (Bazett formula)
SCD: Resuscitated sudden cardiac death
TRPM4: Transient potential melastatin 4 gene
WT: Wild type
Y: Year
